# Parkin Promotes Degradation of the Mitochondrial Pro-Apoptotic ARTS Protein

**DOI:** 10.1371/journal.pone.0038837

**Published:** 2012-07-09

**Authors:** Stav Kemeny, Dikla Dery, Yelena Loboda, Marshall Rovner, Tali Lev, Dotan Zuri, John P. M. Finberg, Sarit Larisch

**Affiliations:** 1 Cell Death Research Laboratory, Department of Biology, Faculty of Sciences, University of Haifa, Mount Carmel, Haifa, Israel; 2 Department of Molecular Pharmacology, The Ruth and Bruce Rappaport Faculty of Medicine, Technion – Israel Institute of Technology, Bat-Galim, Haifa, Israel; The University of Texas MD Anderson Cancer Center, United States of America

## Abstract

Parkinson’s disease (PD) is associated with excessive cell death causing selective loss of dopaminergic neurons. Dysfunction of the Ubiquitin Proteasome System (UPS) is associated with the pathophysiology of PD. Mutations in Parkin which impair its E3-ligase activity play a major role in the pathogenesis of inherited PD. ARTS (Sept4_i2) is a mitochondrial protein, which initiates caspase activation upstream of cytochrome c release in the mitochondrial apoptotic pathway. Here we show that Parkin serves as an E3-ubiquitin ligase to restrict the levels of ARTS through UPS-mediated degradation. Though Parkin binds equally to ARTS and Sept4_i1 (H5/PNUTL2), the non-apoptotic splice variant of *Sept4*, Parkin ubiquitinates and degrades only ARTS. Thus, the effect of Parkin on ARTS is specific and probably related to its pro-apoptotic function. High levels of ARTS are sufficient to promote apoptosis in cultured neuronal cells, and rat brains treated with 6-OHDA reveal high levels of ARTS. However, over-expression of Parkin can protect cells from ARTS-induced apoptosis. Furthermore, Parkin loss-of-function experiments reveal that reduction of Parkin causes increased levels of ARTS and apoptosis. We propose that in brain cells in which the E3-ligase activity of Parkin is compromised, ARTS levels increase and facilitate apoptosis. Thus, ARTS is a novel substrate of Parkin. These observations link Parkin directly to a pro-apoptotic protein and reveal a novel connection between Parkin, apoptosis, and PD.

## Introduction

Parkinson’s disease (PD) is one of the most common progressive neurodegenerative disorders caused by selective degeneration of the dopaminergic neurons in the substantia nigra pars compacta (SNpc) [1], [2]. Loss of function mutations in the *Parkin* gene (PARK 2) are linked to inherited PD and result in neurodegeneration [3], [4]. Several studies have identified mutations of Parkin as risk factors for typical late-onset PD 5,6. Biochemical studies have demonstrated that Parkin has an E3-ubiquitin ligase activity and that mutations in Parkin exhibit loss of E3-ligase function [7]–[10]. E3-ubiquitin ligases promote conjugation of ubiquitin to target proteins for degradation by the Ubiquitin Proteasome System (UPS) [11], [12]. Dysfunction of the UPS was shown to play a major role in the pathophysiology of PD [13]–[15]. Many studies indicate a common theme centered on the role of Parkin in neuroprotection [16]–[20]. Moreover, Rosen et al. showed that the cytoprotective function of Parkin in Alzheimers disease involves the removal of cellular beta-amyloid through a proteasome-dependent pathway 21. Although the exact mechanism by which Parkin protects neurons from degeneration remains largely unknown, accumulating evidence suggests that it involves inhibition of programmed cell death, apoptosis [14], [22], [23].

**Figure 1:**
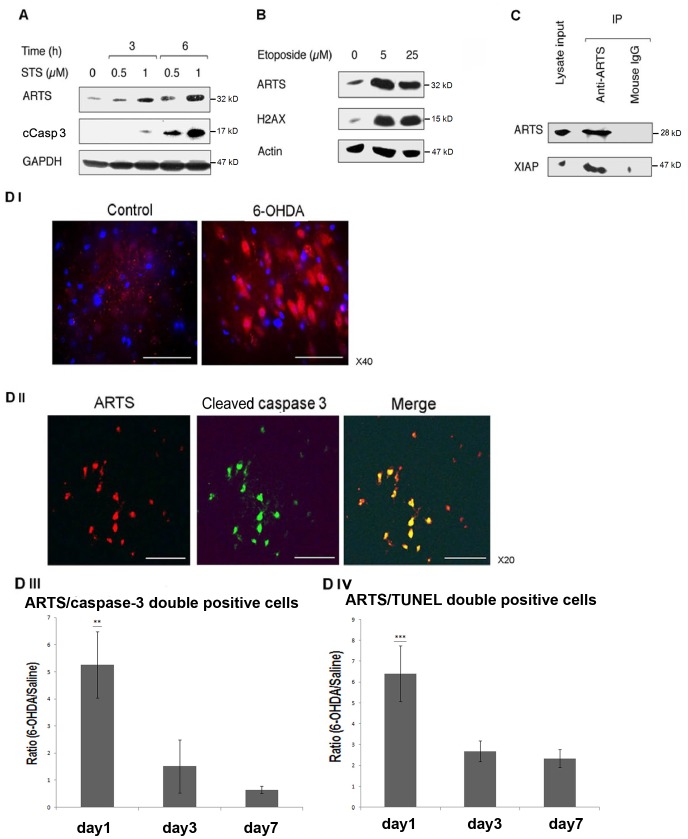
Up-regulation of ARTS levels is associated with induction of apoptosis in SH-SY5Y cells and in 6-OHDA treated rat brains. A. Western blot (WB) analysis shows a strong increase in the expression levels of ARTS upon treatment of SH-SY5Y cells with the apoptotic inducer staurosporine (STS). This elevation in levels of ARTS is associated with an increase in caspase-3 activation. GAPDH was used as a loading control. B. A significant increase in levels of ARTS is seen in response to treatment with etoposide inducing apoptosis in SH-SY5Y cells. This strong up-regulation of ARTS is associated with a corresponding increase in expression of the apoptotic marker H2AX. Actin was used as a loading control. C. Endogenous ARTS binds to XIAP in SH-SY5Y cells. Immunoprecipitation assay (IP) with monoclonal anti ARTS antibody was performed on lysates from SH-SY5Y cells. Mouse IgG served as control for co-precipitation of the specific antigen. DI. A representative picture (viewed with x40 objective) showing immunofluorescence (IF) staining using anti-ARTS antibody in *Substantia nigra pars compacta* (SNpc) of rat brain treated with 6-hydroxy dopamine (6-OHDA) as compared to control injected with saline. 6-OHDA was injected into the left medial forebrain bundle of these rats, from where it is transported to the SN. A significant increase in expression of ARTS is shown in these cells (red). DAPI staining of nuclei is shown in blue. Scale bar represents 50 µm. DII. Co-localization of ARTS (red) and cleaved caspase-3 (green) is seen in a representative rat brain section of 6-OHDA treated rat viewed using x20 objective. Scale bar represents 100 µm. DIII, DIV. Percent of ARTS/cleaved caspase-3 and ARTS/TUNEL double positive cells in SNpc of 6-OHDA treated rats compared to saline injected controls. These pictures demonstrate the levels of ARTS among apoptotic neurons. Counts were done in sections from brains after one, three and seven days following injection of 6-OHDA or saline. Figures present mean ± SEM of the ratio between the percent of double positive cells in 6-OHDA brains relative to the average percent of double positive cells in saline treated brains, at each time point. A significant increase in percentage of ARTS/caspase-3 and ARTS/TUNEL double positive SN neurons is seen 24 hours after injection of 6-OHDA as compared to controls (significance levels were calculated from data shown in Table S1; **P<0.01, ***P<0.001 for difference from same day control by ANOVA with Bonferroni post hoc test). This suggests that ARTS may play an important role in promoting susceptibility to 6-OHDA-induced apoptosis in SN neurons.

Apoptosis is a morphologically distinct form of natural cell death that plays an important role in development and tissue homeostasis, and aberrant apoptosis is associated with a wide variety of diseases and neurodegeneration including PD [24]–[28]. A central step in the execution of apoptosis is the activation of caspases, a family of proteases that are widely expressed as weakly active zymogens. Caspases are regulated by both activators and inhibitors, such as IAPs (Inhibitor of Apoptosis Proteins). XIAP, the most studied and probably the most potent IAP, directly binds to caspases and inhibits their apoptotic activity [23], [29]–[32]. ARTS (Sept4_i2) (henceforth referred to as ARTS) is a mitochondrial pro-apoptotic protein encoded by the *Sept4* gene [33]–[35]. High levels of ARTS are sufficient to induce apoptosis in many cell types [33], [35]–[37]. Conversely, deletion of *Sept4*/ARTS in the mouse leads to increased levels of XIAP, defects in apoptosis and increased tumor development [38], [39]. Although ARTS was originally thought to localize within mitochondria, subsequent work showed that ARTS is localized to the mitochondrial outer membrane (MOM) [40]. In response to apoptotic stimuli, ARTS translocates from the mitochondria to the cytosol where it binds to and inhibits XIAP, leading to caspase activation and cell death [33], [40], [41]. In living cells, ARTS levels are tightly regulated through degradation by the UPS to prevent unwanted cell death [36], [42]. ARTS is a distinct splice variant of the *Sept4* gene [35], [43]. Septins have been traditionally studied for their role in cytokinesis and filament forming abilities and subsequently have been implicated in many other diverse functions [44], [45]. Another Septin, Septin5/hCDCrel1 was shown to serve as a substrate for Parkin [9], [46]. In addition, Sept4_i1 (also known as H5/Pnutl2 [47]) was detected in cytoplasmic proteinaceous inclusions, termed Lewy bodies, these are one of the hallmarks of PD surviving neurons [48]. Importantly, this Septin 4 isoform does not promote apoptosis [33], [49]. These observations raised the possibility that Parkin protects neurons through directly regulating the levels of the pro-apoptotic ARTS protein.

Here we show that in response to pro-apoptotic stimuli, ARTS accumulates in human cultured neuronal-like cells and co-localizes with active caspase-3 and TUNEL staining in degenerating dopaminergic neurons in 6-OHDA injected rat brains which may serve as a model for PD. We show that although Parkin can bind to both isoforms of Septin 4 (ARTS and Sept4_i1), Parkin specifically ubiquitinates and degrades ARTS, but not Sept4_i1 through proteasome-mediated degradation. Since Sept4_i1 does not promote apoptosis [33], [36], it appears that the binding and degradation of ARTS by Parkin is specific and related to the pro-apoptotic function of ARTS. Moreover, Parkin loss-of-function experiments reveal that reduction of Parkin causes increased levels of ARTS and apoptosis. This suggests that neurons of PD patients with mutations in Parkin that impair its E3-ligase function, may accumulate increased levels of ARTS and therefore have increased susceptibility to neuronal cell death. Although a variety of substrates have been identified for Parkin, ARTS, which has a direct known role in initiating apoptosis, provides a new explanation for the neuroprotective activity of Parkin and reveals a novel connection between Parkin, apoptosis, and PD. Furthermore, our data suggest that ARTS is a potential new target for developing treatments against PD.

**Figure 2:**
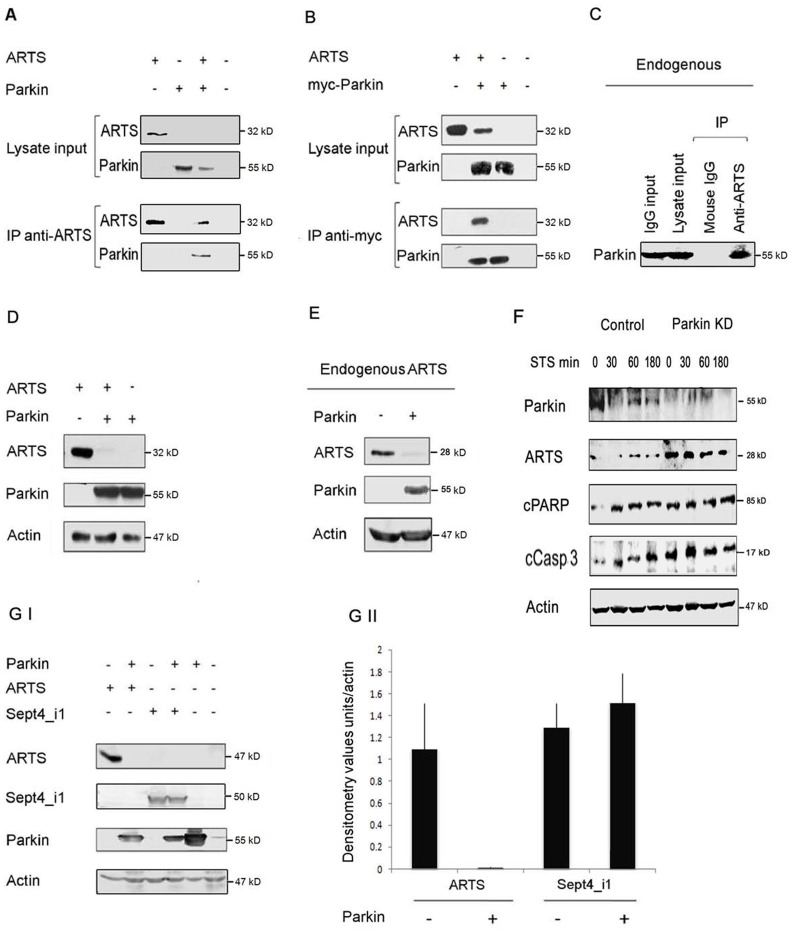
Parkin binds to ARTS and reduces its expression levels through the Ubiquitin-Proteasome System (UPS). A.B. Both exogenous and endogenous Parkin and ARTS bind to each other. COS-7 cells were co-transfected with AU5-ARTS (32 kD) and myc-Parkin (52 kD). Lysates were subjected to IP with anti-ARTS (I) and anti-myc (II) and then to WB analysis. These results show that exogenous ARTS and Parkin can bind to each other. **C**. Lysates of MEFs were subjected to IP with monoclonal anti-ARTS antibody. These results show that endogenous ARTS and Parkin can bind to each other. **D.E.** Parkin down-regulates exogenous and endogenous expression levels of ARTS. **D**. The expression levels of transfected AU5-ARTS and Parkin were determined in COS-7 cells, which contain low levels of endogenous ARTS. Lysates were subjected to WB analysis with monoclonal anti-ARTS and anti-Parkin antibodies. Transfection of 1myc-Parkin dramatically reduces the levels of ARTS in these cells. **E.** Endogenous levels of ARTS were determined in HeLa cells, which contain relatively high endogenous levels of ARTS. Lysates were subjected to WB analysis with monoclonal anti-ARTS and anti-Parkin antibodies. Transfection of Parkin dramatically reduces the endogenous levels of ARTS in these cells. **F.** Parkin knocked -down SH-SY5Y cells exhibit increased levels of ARTS and elevated apoptosis. A stable Parkin knockdown (Parkin KD) cell line of neuroblastoma SH-SY5Y in which Parkin expression was knocked down by short hairpin RNAs (shRNAs) was established. These Parkin KD cells were treated with 1.5 µM of STS for the indicated time periods. These Parkin KD cells exhibited elevated levels of ARTS and increased levels of apoptosis as determined by increased levels of two different markers of apoptosis, cleaved caspase-3(cCasp3), and its substrate, cleaved PARP (cPARP). **GI, II.** Parkin specifically reduces the levels of ARTS but not the non-apoptotic Sept4_i1 splice variant. COS-7 cells were co-transfected with 1myc-Parkin together with Flag-Sept4_i1 or 6myc-ARTS. The levels of ARTS but not Sept4_i1 were strongly down-regulated by Parkin. **GII.** Densitometry analysis of Western blot results shown in Fig GI. Protein levels were normalized with respect to actin. Graph presents mean ± SEM of the densitometry values relative to actin levels.

## Methods

### 6-OHDA Rat PD Model

Protocols for animal experiments were approved by the Technion Institutional Animal Care and Use Committee. The dopaminergic neurotoxin 6-hydroxydopamine (6-OHDA, 8 µg) or saline (control) were injected to the left medial forebrain bundle of Sprague-Dawley male rats one, three or seven days previous to whole-body perfusion fixation with paraformaldehyde. Coronal cryostat sections (30 μ) were stained with TUNEL reagent or by immunofluorescence (IF) assay using polyclonal anti-ARTS or monoclonal anti-cleaved caspase-3 as primary antibodies [50]. Sections were viewed using a UV microscope equipped with high-resolution digital camera, and photomicrographs were made of the substantia nigra pars compacta area (identified by presence of large dopaminergic cell-bodies) at a level corresponding to bregma -4.8–4.9 according to previously ascertained topographical markers. Numbers of ARTS, merged ARTS-cleaved caspase-3, or merged ARTS-TUNEL positive cells, were counted from 1–3 pictures from each of 3–4 sections from each rat and the data was expressed as a percentage of total DAPI-positive nuclei in the microscope field. The data was therefore taken mainly from substantia nigra pars compacta but also including a small percentage of cells from an area immediately adjacent to it.

### Cell Cultures

COS-7, HEK293 and HeLa cell lines were grown in Dulbecco’s Modified Eagle Medium (DMEM) with 4.5 g/l D-glucose. SH-SY5Y cells were grown in DMEM/F-12. Media were supplemented with 10% fetal bovine serum (FBS), penicillin- streptomycin 100 U/ml, sodium pyruvate 1 mM and L-glutamine 2 mM (Biological Industries, Israel). MEFs were isolated from 12.5 days mouse embryos and used through the fifth passage. Cells were grown in DMEM supplemented as above. Etoposide and staurosporine (STS) were purchased from Sigma. MG132 was purchased from Alexis and was applied at a concentration of 20 µM for 6 hours.

**Figure 3:**
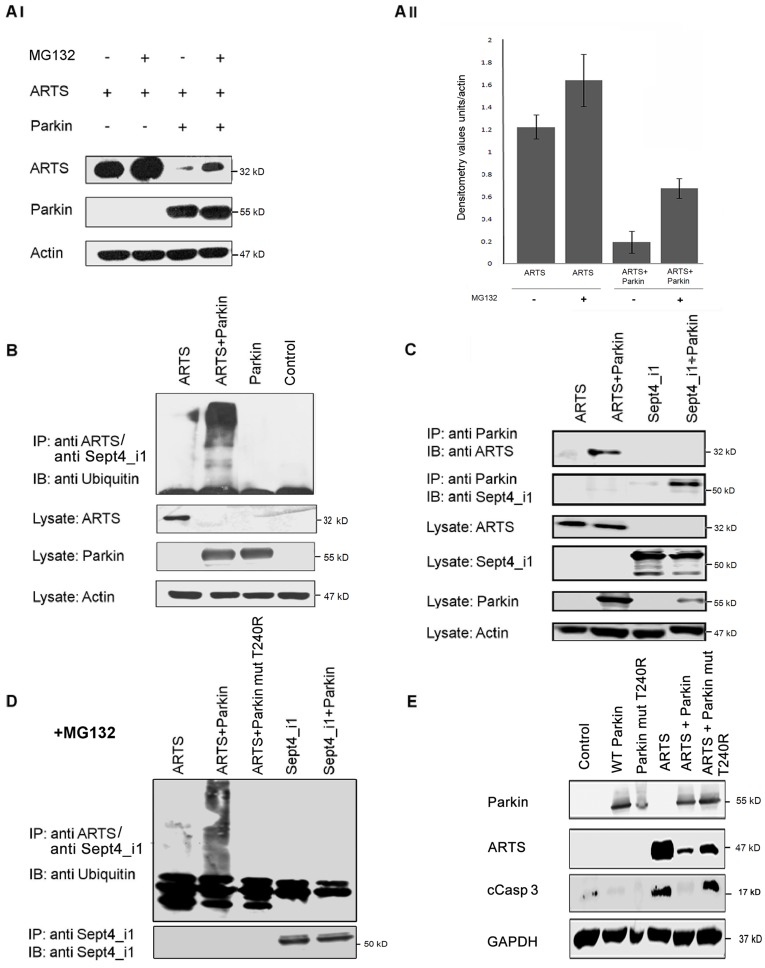
Although Parkin can interact with both ARTS and Sept4_i1, it selectively ubiquitinates only ARTS. AI, II . Parkin promotes degradation of ARTS through the Ubiquitin Proteasome System (UPS). COS-7 cells co-transfected with 1myc-Parkin and AU5-ARTS were treated with the proteasome inhibitor, MG132. Expression levels of ARTS were significantly decreased in the presence of Parkin, and were restored upon addition of MG132. **AII.** Densitometry analysis of WB results shown in AI. Protein levels were normalized to actin. Graph presents mean ± SEM of the densitometry values relative to actin levels. **B.** Parkin serves as an E3-ligase for ARTS. COS-7 cells co-transfected with 1myc-Parkin, AU5-ARTS and HA-8xubiquitin were subjected to in vivo ubiquitination assay followed by immunoprecipitation (IP) with either monoclonal anti-ARTS antibody or anti-Sept4_i1 antibody. Non-transfected cells and cells transfected with only 1myc-Parkin or AU5-ARTS served as controls. ARTS is ubiquitinated in the presence of Parkin. **C.** Parkin binds both ARTS and Sept4_i1. COS-7 cells were co-transfected with 1myc-Parkin together with Flag-Sept4_i1 or AU5-ARTS. Lysates were subjected to IP with anti-Parkin antibody followed by WB analysis. **D.** COS-7 cells treated with the proteasome inhibitor, MG132 were co-transfected with 1myc-Parkin or mutant Parkin T240R together with 6myc-ARTS or Flag -Sept4_i1. Immunoprecipitation was done with either anti-ARTS or anti-Sept4_i1 antibody followed by *In vivo* ubiquitination assay. Western blot analysis was performed with anti-ubiquitin antibody (see methods). Parkin selectively ubiquitinates ARTS but not Sept4_i1. Mutant T240R Parkin lacking E3-ligase activity, demonstrates markedly decreased ability to ubiquitinate ARTS as compared to wt Parkin. **E**. COS-7 cells were transiently transfected with ARTS, Parkin, and the mutant Parkin T240R with compromised E3-ligase activity. The levels of cleaved caspase-3 (cCasp3) representing rate of apoptosis were visualized using western blot analyses. Co-transfection of COS-7 cells with ARTS and Parkin resulted in ARTS degradation and apoptotic suppression, as visualized by the absence of cleaved caspase-3 (cCasp3). In contrast, co-transfection with ARTS and mutant Parkin T240R restored apoptosis in these cells.

### Generation of Stable Parkin-KD SH-SY5Y Cells

To generate SH-SY5Y cells stably expressing Parkin shRNAs, subconfluent cultures were co-transfected with shRNA1 (NM_013988.X_2399S1C1, Sigma) and shRNA3 (NM_013988.X_425S1C1, Sigma). As a negative control MISSION® PLKO.1puro empty vector (SHC001, Sigma) was used. Nucleofection of SH-SY5Y cells was performed using the G-04 program of Nucleofactor (AMAXA), as per manufacturer’s instructions. Following selection with 0.6 mg/ml Puromycin (Sigma), mass cultures were tested for reduction of endogenous Parkin. These cultures were used in this study.

### Generation of Transient Parkin-KD SH-SY5Y Cells

To generate SH-SY5Y cells transiently expressing Parkin siRNA, subconfluent cultures were transfected with Stealth Select RNAiTM siRNA (Park2, oligo ID HSS107595, Invitrogen). As a negative control Stealth RNAiTM siRNA Negative Control Hi GC (12935-400, Invitrogen) was used. Transfection was performed with LipofectamineTM 2000 Reagent (Invitrogen) according to the manufacturer’s instructions.

**Figure 4:**
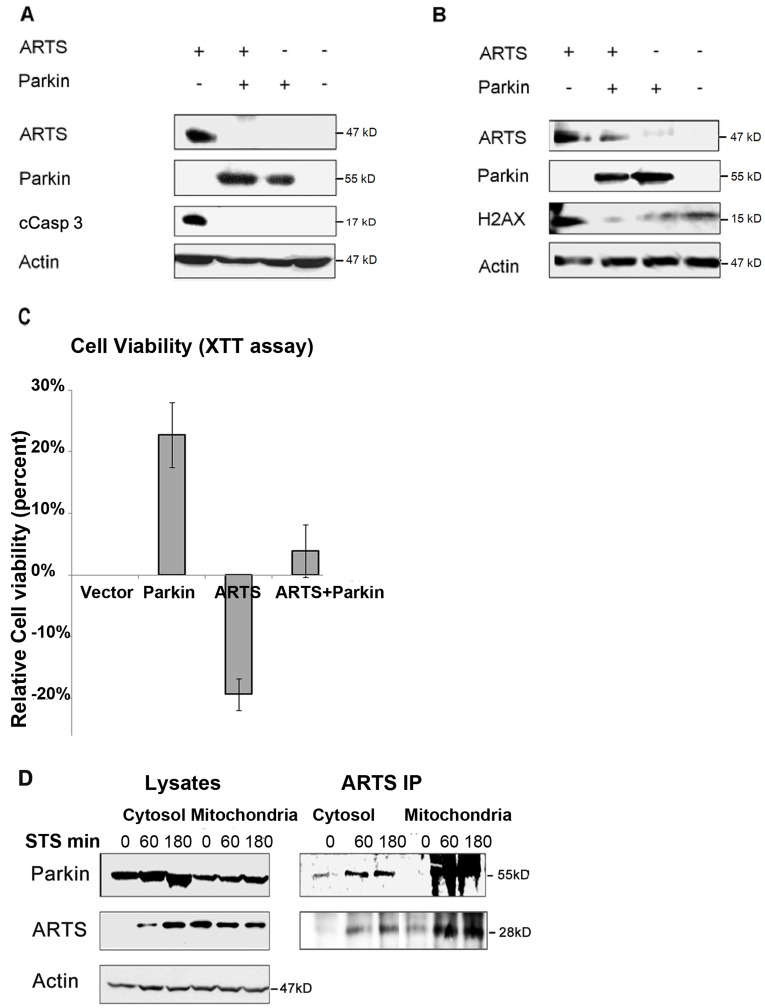
UPS mediated degradation of ARTS by Parkin protects cells from apoptosis. **A. and B.** Parkin inhibits apoptosis induced by ARTS. COS-7 cells were co-transfected with 1myc-Parkin and 6myc-ARTS. Over-expression of ARTS alone was sufficient to induce apoptosis in these cells. Apoptosis is exhibited by the presence of the apoptotic markers cleaved caspase-3 (A) and H2AX (B). Thus, overexpression of Parkin can strongly inhibit ARTS-induced apoptosis. **C.** Co-transfection of 1myc-Parkin and AU5-ARTS confers a protective effect against cell death. Cell viability was quantified using XTT-based assay (see Materials and Methods). The results are represented as a ratio between each transfection and the negative control (mean± SE, n = 6). The viability of cells transfected with empty vector was defined as the baseline viability of the assay. Cell viabilities were recorded in relation to this baseline. **D.** ARTS binds to Parkin both at the mitochondria and in the cytosol. Sub cellular fractionation was performed using 140 µg/ml digitonin. Apoptosis was induced using 1.5 µM STS for 2 h. Immunoprecipitation (IP) of ARTS from both cytosolic and mitochondrial fractions revealed basal binding of ARTS to Parkin under non apoptotic as well as under apoptotic conditions.

### Constructs

pSC2-6myc ARTS (47 kD) and pEF1-AU5-ARTS (32 kD) were generated using PCR. 6myc and AU5 tags are attached to the N-terminus of ARTS. The 1myc Parkin construct was a kind gift from Prof. Simone Engelender (Molecular Pharmacology Dept., Rappaport Faculty of Medicine, Technion, Israel). HA-8Xubiquitin construct was a kind gift from Prof. Aaron Ciechanover (Molecular Pharmacology Dept., Rappaport Faculty of Medicine, Technion, Israel). pEYFP-C1 Parkin was a kind gift from Prof. Richard J. Youle (National Institutes of Health, Bethesda, MD, USA). The mutant Parkin T240R construct was a kind gift from Prof. Mia Horowitz.

### Cell and Tissue Lysis

Cells and frozen mice tissues were lysed in RIPA buffer: 150 mM NaCl, 50 mM Tris–HCl (pH 8), 1% NP–40, 0.5% Na-deoxycholate, 0.1% SDS with protease inhibitors (Complete, Roche Applied Science). For IP experiments less stringent lysis buffer was used: 150 mM NaCl, 50 mM Tris–HCl (pH 8), 1% NP–40, 0.5% Na-deoxycholate. The lysates were incubated on ice for 30 min and then centrifuged at 15,000× g for 25 min at 4°C. The supernatant was collected and protein concentration was determined using the Micro-BCA kit (Pierce).

**Figure 5:**
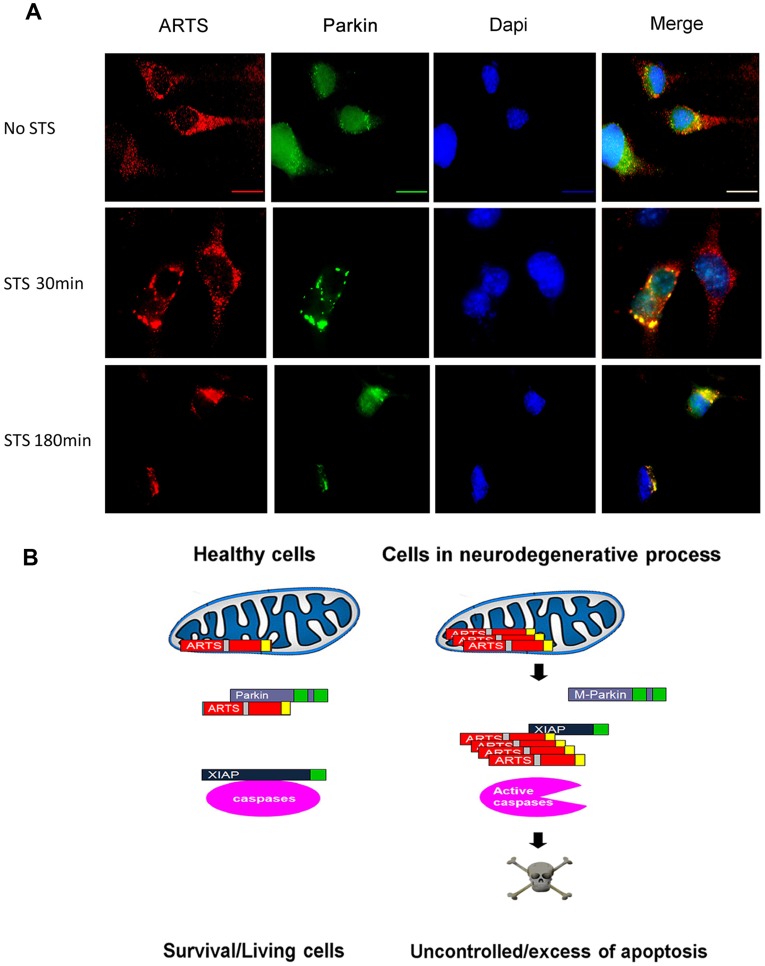
ARTS and Parkin exhibit co-localization in SH-SY5Y cells. **A.** Immunofluorescence (IF) assay was performed on SH-SY5Y cells, which were transiently co-transfected with AU5-ARTS (red) and 1myc-Parkin (green) and treated with 1.5 µM STS. These IF results reveal co-localization of ARTS and Parkin in untreated cells, with increased co-localization as early as 30 min after apoptotic induction. **B.** Working model for the role of ARTS in healthy Parkin expressing cells, and in cells containing mutated or dysfunctional Parkin. We propose that in healthy brain cells which contain intact Parkin, the levels of ARTS are kept low through constant down regulation by Parkin. Upon initiation of a stress or apoptotic stimuli, recruitment of Parkin to dysfunctional mitochondria occurs as well as translocation of ARTS to the cytosol at the early stages of apoptosis [40]. This may allow the binding and degradation of ARTS by Parkin and help maintain low levels of ARTS. This down-regulation of ARTS is expected to promote cell survival by preventing the inhibition of XIAP and caspase activation [33], [40]. According to our model, in brain cells containing mutated or dysfunctional Parkin, Parkin can no longer degrade ARTS. This leads to accumulation of ARTS and initiation of apoptosis through de-repression of caspases by antagonizing XIAP. This first wave of caspases leads to Mitochondrial Outer membrane Permeabilization (MOMP) which results in release of mitochondrial factors residing in the inner membrane space (IMS) such as Cytochrome c and Smac/Diablo, causing amplified caspase activation and cell death [40].

### Antibodies

Antibodies to the various proteins were purchased from the indicated companies, and used as instructed. Importantly, unless noted otherwise, in all our assays we used the monoclonal or polyclonal anti-ARTS antibodies (Sigma, St. Louis) which are the only currently commercially available antibodies directed against the unique C-terminus of ARTS. Other sources of antibodies were: against Parkin (sc-32282, Santa Cruz), XIAP (#610716, BD), H2AX (DR1016, Calbiochem), cleaved caspase-3 (#9661, Cell Signaling), β-actin (#69100, MP Biomedicals) and matching secondary antibodies from Jackson laboratories.

### Protein Reactions and other Experimental Procedures

Procedures for WB, IP and *in vivo* ubiquitination are done as previously described [36].

### Densitometry Analysis

Densitometry analyses of Western blot results were performed using TotalLab TL100 graphic software.

### XTT

The XTT assay was performed to study the effect of Parkin and ARTS on cell viability. The assay was performed in 96-well plate format according to directions of the supplier (Biological Industries Ltd.). HeLa cells were transfected with 100 ng ARTS, or Parkin or ARTS together with Parkin. 24 hours post transfection 50 µl of XTT solution was added to each well and plates were incubated at 37°C in 5% CO2 for 2 h. Absorbance of the water-soluble formazan salt was measured on PowerWaveXS Elisa reader at primary wave length of 450 nm and a reference wave length of 630 nm. Empty vector was used as a negative control. The assay was performed in 6 replicates. The viability of cells transfected with empty vector was defined as baseline. Relative cell viability was calculated using the following formula:
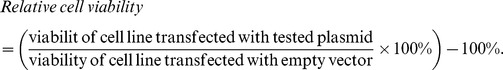



### Immunofluorescence Assay

Cells were transiently transfected with AU5-ARTS and 1myc- Parkin in 24well plates. Cells were seeded on cover glasses previously coated with fibronectin (5 µg/ml). Following apoptosis induction (STS 1.75 µM) cells were fixed with 4% paraformaldehyde in PBS for 20 min at room temperature and washed with PBS. Image analysis was carried out using fluorescent microscopy (Nikon 50i).

### Cell Fractionation by Digitonin

Following induction of apoptosis, cytosolic fraction was generated using a digitonin-based subcellular fractionation technique. Briefly, cells were harvested and centrifuged at 300 g for 10 min, washed in PBS and repelletted. Cells were permeabilized for 5 min on ice with cytosolic extraction buffer (250 mM sucrose, 70 mM KCl, 137 mM NaCl, 4.3 mM Na2HPO4, 1.4 mM KH2PO4 pH 7.2, protease inhibitor cocktail (Complete, Roche)) containing freshly prepared digitonin (140 µg/ml, D-5628, Sigma). Cytosolic fraction was isolated by collecting the supernatant after centrifugation at 1000 g for 5 min at 4°C.

### In vivo Ubiquitination and Immunoprecipitation Assay

Cells were transiently transfected with different constructs together with HA-8xubiquitin construct. Immunoprecipitation was performed with the indicated antibodies. In vivo ubiquitination assays were performed as previously described in (36, 64).

### Statistics

For the in vivo rat data, mean cell counts for 6-OHDA-treated and corresponding control animals were compared using one-way ANOVA. Correlations between continuous variables (days, densitometry values) were calculated using Pearson’s correlation coefficient.

## Results

### Up-regulation of ARTS Levels is Associated with Induction of Apoptosis in SH-SY5Y Cells and in 6-OHDA Treated Rat Brains

High levels of ARTS are sufficient to promote apoptosis in a large number of cell lines [33], [36]. Moreover, COS-7 cells stably transfected with an ARTS plasmid under the control of an inducible promoter, resulted in induction of apoptotic cell death when ARTS levels were increased [36]. ARTS promotes caspase activation through binding and antagonizing XIAP [33], [40]. Furthermore, a significant increased resistance to cell death was seen both in cells in which the expression of ARTS was knocked-down and in hematopoietic stem and progenitor cells from *Sept4*/ARTS-null mice [38], [40]. This provides a compelling evidence for the physiological role of ARTS in promoting apoptosis *in vivo*. We have previously shown that overexpression of ARTS in CAD (human CNS catecholaminergic cells which underwent differentiation into mature neurons), resulted in strong activation of caspases and apoptosis [51]. Here we show that treatment of SH-SY5Y human neuroblastoma-derived neuronal cells with various pro-apoptotic stimuli such as staurosporine (STS), and etoposide leads to a significant increase in endogenous levels of ARTS (Figs. 1A–C). SH-SY5Y cells express dopamine receptors and the noradrenaline-synthesizing enzyme dopamine β-hydroxylase [52]. Thus, they resemble other CNS catecholaminergic neurons such as dopaminergic neurons. Apoptotic induction in these cells as determined by the appearance of the apoptotic markers cleaved caspase-3 and H2AX, resulted in elevated levels of ARTS (Figs. 1A–B). In addition, we show that endogenous ARTS can bind to XIAP in SH-SY5Y cells (Fig. 1C) suggesting that ARTS can promote apoptosis in these neuronal-like cells directly through binding and antagonizing XIAP. Similarly, we observed a strong increase in levels of ARTS in substantia nigra of rat brains injected with the neurotoxic compound 6-OHDA. Figure1D shows a representative section from the SN pars compacta area of a tested rat brain. Immunofluorescence detection using anti-ARTS antibody shows an increased expression of ARTS in the SN pars compacta area of treated rat brains as compared to controls. This increase in ARTS staining is seen despite a gradual decrease in numbers of ARTS-positive neurons over the 7-day observation period by comparison with the controls, caused by the progressive cell death in this period. Importantly, a significant five times increase in percentage of ARTS/cleaved caspase-3 double positive neurons and more than six times increase in percentage of ARTS/TUNEL double-positive cells was seen after one day of 6-OHDA treatment as compared to saline control (Figs. 1DII, III, IV, Table S1). The numbers of double-labeled cells decreased after the first day, as a result of the progressive cell death in this period. These results suggest that ARTS can play an important role in promoting susceptibility to 6-OHDA-induced apoptosis in dopaminergic cells and neurons which simulates the neurodegeneration of PD.

### Parkin Binds to ARTS and Specifically Reduces its Levels through the Ubiquitin-Proteasome System (UPS)

Since high levels of ARTS can promote apoptosis, a tight regulatory mechanism keeping ARTS levels low is important to prevent unwanted cell death. We have shown that in healthy cells the protein levels of ARTS are kept low through constant down-regulation by the UPS [36], [42]. In living cells, ARTS is localized at the outer membrane of mitochondria [33], [35]. Upon induction of apoptosis, ARTS translocates to the cytosol where it accumulates as a result of inhibition of protein degradation by the UPS [33], [36]. High levels of ARTS bind and antagonize XIAP in the cytosol, promoting caspase activation and cell death [33], [36], [41]. To explore the potential role of ARTS in PD we concentrated on investigating the biochemical interaction between ARTS and Parkin. For this purpose, COS-7 cells which are characterized by low levels of endogenous ARTS and Parkin were co-transfected with ARTS and Parkin constructs. Immunoprecipitation of ARTS revealed that these two proteins bind to each other (Figs. 2A, 2B). Moreover, immunoprecipitation assay using specific anti-ARTS antibody on lysates of primary cultured MEFs, showed that endogenous Parkin and ARTS proteins bind to each other as well (Fig. 2C). Importantly, a strong down regulation in ARTS levels was seen when Parkin was co-transfected with ARTS in COS-7 cells (Fig. 2D). Similarly, a strong decrease in endogenous ARTS was seen following transfection of Parkin into HeLa cells which contain relatively high levels of endogenous ARTS (Fig. 2E). Though Sept4_i1 (H5/Pnutl2) shares a relatively high sequence homology with ARTS, it does not promote apoptosis [33], [49]. Interestingly, we have found that Parkin specifically reduces the levels of ARTS but not of Sept4_i1 (Figs. 2GI, II). This suggests that the effect of Parkin on ARTS is specific and related to its pro-apoptotic function. To investigate the effect of loss-of -function of Parkin on levels of ARTS and apoptosis, we established a stable Parkin knockdown (Parkin KD) cell line of neuroblastoma SH-SY5Y in which parkin expression was knocked down by short hairpin RNAs (shRNAs). These Parkin KD cells exhibited elevated levels of ARTS and increased levels of apoptosis as determined by increased levels of two different markers of apoptosis, cleaved caspase-3, and its substrate, cleaved PARP (Fig. 2F). Similar results were obtained using transient expression of siRNAs towards Parkin in SH-SY5Y cells (data not shown). These results indicate that Parkin affects apoptosis through regulating the levels of ARTS.

### Parkin Can Interact with Both ARTS and Sept4_i1 But Ubiquitinates Only ARTS

Parkin has an E3-ubiquitin ligase activity [8], [9]. We have demonstrated that high levels of Parkin selectively reduce the levels of ARTS (Figs. 2GI, II). To further explore whether ARTS serves as a substrate of Parkin, we first used the proteasome inhibitor MG132 to treat COS-7 cells co-transfected with Parkin and ARTS. Significantly, treatment with the proteasome inhibitor, MG132 restored the levels of ARTS which were reduced by Parkin (Fig. 3A). Thus, Parkin degrades ARTS in a proteasome-dependent manner. Next, an *in vivo* ubiquitination assay performed in COS-7 cells co-transfected with Parkin, HA-8xubiquitin and ARTS showed the appearance of ubiquitin-conjugated ARTS in Parkin transfected cells (Fig. 3B). Similar results showing ubiquitination of endogenous ARTS were seen in HeLa cells transfected with Parkin as compared to control (data not shown). To further understand the distinct effect of Parkin on ARTS but not on Sept4_i1, we examined whether Parkin selectively binds to ARTS. We found that ARTS and Sept4_i1 could be equally immunoprecipitated with the anti-Parkin antibody (Fig. 3C). Importantly, though both ARTS and Sept4_i1 are splice variant products of the *Septin 4* gene, ARTS contains different and unique sequences which are responsible for its pro-apoptotic function [33], [37], [41]. Next, we investigated whether Parkin can equally ubiquitinate both these isoforms of the *Septin 4* gene. Using two different proteasome inhibitors (MG132 and Lactacystin) we showed that both inhibitors allowed accumulation of ubiquitinated forms of ARTS when overexpressed with Parkin (Figs 3D and Fig. S1, respectively). Furthermore, using both proteasome inhibitors, immunoprecipitation with either anti-ARTS or anti-Sept4_i1 followed by *in vivo* ubiquitination assay also revealed that although Parkin binds both to ARTS and Sept4_i1, it selectively ubiquitinates ARTS but not the non-apoptotic isoform Sept4_i1 (Fig. 3D, 3E, Fig. S1). Moreover, a mutant form of Parkin (T240R), with a compromised E3-ligase activity [53], could not ubiquitinate ARTS (Fig. 3D). Co-transfection of COS-7 cells with ARTS and Parkin resulted in degradation of ARTS and inhibition of apoptosis, as visualized by the absence of cleaved caspase-3 (cCasp3) (Fig. 3E). In contrast, co-transfection with ARTS and T240R mutant Parkin restored apoptosis in these cells (Fig. 3E). This confirms that the E3-ligase activity of Parkin is required for the degradation of ARTS and for cell survival. Together, these results suggest that Parkin specifically ubiquitinates and degrades ARTS, and that this degradation may be linked to the pro-apoptotic function of ARTS.

### UPS Mediated Degradation of ARTS by Parkin Protects Cells from Apoptosis

We have shown that cells in which ARTS expression is knocked-down, as well as hematopoietic cells from ARTS/Sept4 knockout mice, exhibit a significant increase in their resistance to pro-apoptotic stimuli [38], [40], [54]. To test whether the strong decrease in the levels of ARTS upon over-expression of Parkin affects responsiveness to apoptosis, we examined COS-7 cells co-transfected with Parkin and ARTS. Overexpression of ARTS alone was sufficient to induce apoptosis in these cells, as seen by appearance of two apoptotic markers, cleaved caspase-3 (cCasp3) and H2AX (Figs. 4A, B). In addition, we observed reduced cell viability, as visualized by the XTT assay, under these conditions (Fig. 4C). On the other hand, overexpression of Parkin inhibited ARTS-mediated caspase cleavage and cell death (Fig. 4A, B). To determine the cellular localization in which the interaction between ARTS and Parkin takes place, we performed cell fractionation assays followed by immunoprecipitation (IP) with monoclonal anti ARTS antibodies. These experiments revealed basal binding of ARTS to Parkin under non-apoptotic conditions with increased binding in both the mitochondrial and cytosolic fractions following induction of apoptosis with STS (Fig. 4D). Similarly, immunofluorescence (IF) assays revealed some co-localization of ARTS and Parkin in the untreated cells, and a significant increased co-localization as early as 30 minutes after apoptotic induction in SH-SY5Y cells (Fig. 5A) and in HeLa cells (Fig S2). Collectively, these results suggest that binding of ARTS to Parkin can occur both at mitochondria and in the cytosol (Fig. 4D, 5A and Fig. S2). Taken together, these results are consistent with our model that Parkin promotes cell survival by down-regulating the pro-apoptotic IAP-antagonist ARTS [33], [40].

Based on these results, we propose a working model for the regulation of apoptosis by Parkin and ARTS (Fig. 5B). We propose that in healthy brain cells which contain intact Parkin, the levels of ARTS are kept low through constant down regulation by Parkin. Upon initiation of a stress or apoptotic stimuli, recruitment of Parkin to dysfunctional mitochondria occurs as well as translocation of ARTS to the cytosol at the early stages of apoptosis [40]. This may allow the binding and degradation of ARTS by Parkin and help maintain low levels of ARTS. This down-regulation of ARTS is expected to promote cell survival by preventing the inhibition of XIAP and caspase activation [33], [40]. According to our model, in brain cells containing mutated or dysfunctional Parkin, Parkin can no longer degrade ARTS. This leads to accumulation of ARTS and initiation of apoptosis through de-repression of caspases by antagonizing XIAP. This first wave of caspases leads to Mitochondrial Outer Membrane Permeabilization (MOMP) which results in release of mitochondrial factors residing in the inner membrane space (IMS) such as Cytochrome c and Smac/Diablo, causing amplified caspase activation and cell death [40].

## Discussion

Parkinson’s disease is characterized by selective degeneration of the dopaminergic neurons in the substantia nigra pars compacta (SNpc) occurring by apoptosis [1], [2]. Here we show that rat brains treated with 6-OHDA exhibit a significant increase in expression of ARTS in the SNpc together with increased levels of apoptosis (cleaved caspase-3 and TUNEL) in these neurons (Figs. 1DIII, IV, Table S1). The effects of 6-OHDA treatment mimic the behavioral and biochemical characteristics of PD in the nigro-striatal dopaminergic system [55]. In addition, 6-OHDA treatment is marked by the apoptotic death of dopaminergic neurons in the SNpc. Moreover, this treatment is characterized by a rapid course of cell death seen over the first week after injection [56], [57]. Our results showing significant increase in ARTS-positive -apoptotic cells seen mainly within 24 hours following 6-OHDA treatment, are consistent with the caspase-3 cleavage data in MPTP mice model for PD, which reveal that caspase-3 cleavage peaks early, at days 1 and 2 after the end of MPTP intoxication in these mice [58]. These results suggest that ARTS can play an important role at the initiation stages of 6-OHDA-induced-apoptosis and can promote susceptibility to 6-OHDA-induced apoptosis which simulates neurodegeneration processes in PD.

There are two major protein splice variants of the *Sept4* gene; Sept4_i1 [H5, pnutl2 [47]] and Sept4_i2/ARTS [35], [43]. ARTS is the only splice variant of the Sept4 locus which was shown to directly promote apoptosis [33], [49]. Here we demonstrate that although Parkin binds equally to both ARTS and Sept4_i1 (Fig. 3C), it specifically and selectively ubiquitinates and reduces the levels of ARTS but not of the non-apoptotic isoform Sept4_i1 (Figs. 2GI, II, 3D, Fig S1). This indicates that the effect of Parkin on ARTS is specific and related to its pro-apoptotic function. Of note, although Sept4_i1 was reported to be a substrate for Parkin [54], no experimental data to support this statement are available in the published literature. Several substrates of Parkin have been described, e.g. Sept5/CDCrel1 [46], glycosylated α-Synuclein [59] Pael-R [60], synphilin-1 [61], Eps15 [62], the p38 subunit of the aminoacyl-tRNA synthetase complex [63] mutant glucocerebrosidase (GCase) [64] and PICK1 [65]. None of them was shown to play a direct role in apoptosis. Thus, ARTS which can directly promote apoptosis seems to be a substrate of Parkin. We have recently reported that XIAP also acts as an E3-ligase for ARTS [42]. It is not unusual that multiple E3 ligases contribute to the stability of substrates as, for example, at least five different E-ligases have been shown for p53 [66]–[70]. Therefore, it is reasonable that under different conditions and in different tissues either XIAP, Parkin or both regulate the levels of ARTS to prevent unwanted apoptosis. The Sept4 protein Sept4_i1 was found to co-localize with alpha-synuclein in Lewy bodies, containing abnormal protein aggregations, typical of neuronal degeneration associated with PD [48]. Interestingly, alpha-synuclein is ubiquitylated in Lewy bodies by the ubiquitin-protein ligase seven in absentia homolog (SIAH) leading to its aggregation, which is toxic to cells [71]. Garrison et al, has shown that ARTS directly binds to SIAH [72]. It is therefore possible, that ARTS may be involved in these aggregates leading to dopaminergic cell death [73]. Importantly, Shehadeh et al. report a more than 10-fold increase in expression of Sept4 protein in postmortem PD brain samples versus controls [54]. Since this study was done using antibodies which detect both isoforms of *Sept4* (directed at their shared N-terminus), it is possible that ARTS is responsible for some, if not all of the observed increase in Sept4 levels in these PD samples.

There have been many reports of apoptosis in postmortem PD brains [reviewed in [74]. Studies performed on post mortem samples from PD patients and in different *in vivo* and *in vitro* systems, reveal that PD pathogenesis involves the mitochondrial intrinsic apoptotic pathway [75]. ARTS plays an important physiological role in this pathway [33], [38], [72]. In living cells, the majority of ARTS is localized at the mitochondrial outer membrane (MOM) [40]. Parkin was found in the cytosol and at mitochondria [17], [76]–[79]. Significantly, under stress conditions, Parkin translocates to mitochondria and ubiquitinates substrates on the mitochondrial surface, targeting them for degradation [80].

The cellular localization of ARTS and Parkin at the mitochondrial surface may explain the differential ubiquitination and degradation of ARTS, but not the non-apoptotic splice variant Sept4_i1. Whereas ARTS is localized at the (MOM) [40], Sept4_i1 localizes to actin filaments [81]. It is therefore possible that a functional complex forms only between Parkin and ARTS at the MOM, but not with Sept4_i1 which is located in a different cellular compartment. Recently, it was shown that Parkin is selectively recruited to dysfunctional mitochondria and subsequently promotes their autophagic degradation [82]–[84]. Moreover, Narendra et al. have found that PINK1, a putative kinase mutated in autosomal recessive forms of PD, signals mitochondrial dysfunction to Parkin, and that PINK1 accumulation on mitochondria is both necessary and sufficient for Parkin recruitment to mitochondria and induction of mitophagy [84]. Our fractionation, IP and IF experiments show that ARTS can bind to Parkin both in the cytosol and at the mitochondria (Fig. 4D, 5A and Fig S2A,B,C). We therefore suggest that recruitment of Parkin to dysfunctional mitochondria as well as translocation of ARTS to the cytosol at the early stages of apoptosis [40] may allow the binding and degradation of ARTS by Parkin and help maintain low levels of ARTS. This down-regulation of ARTS is expected to promote cell survival by preventing the inhibition of XIAP and caspase activation [33], [40].

Interestingly, based on the neuroprotective role of Parkin, Ulusoy and Kirik suggest that overexpression of Parkin can provide a novel strategy for neuroprotection in PD [85]. Our results support this approach and provide an additional insight into the mechanism of neuroprotection by Parkin. In addition, other mechanisms by which loss of Parkin function promotes caspase activation have been reported. For example, da Costa et al., reported that Parkin significantly decreased caspase-3 activity and p53 expression in 6-OHDA-treated SH-SY5Y cells [86]. It is also worth noting that caspases play non-apoptotic roles during cellular remodeling, including dendritic pruning, and that elevated caspase activity has been implicated in axonal degeneration in Alzheimer’s Disease (see for example, [87]–[90]). Therefore, it is possible that defects in Parkin-mediated degradation of ARTS may also lead to increased but non-lethal levels of caspase activity that causes neurite degeneration. This may be particularly relevant during the early stage of PD, prior to the onset of widespread cell death [91]–[93]. Collectively, we show that ARTS is a novel substrate of Parkin. Furthermore, our work provides a new explanation for the neuroprotective activity of Parkin and reveals a potentially direct link between Parkin, apoptosis and PD.

## Supporting Information

Figure S1 **Parkin selectively ubiquitinates ARTS but not Sept4_i1.** COS-7 cells were co-transfected with 1myc-Parkin together with 6myc-ARTS or Flag -Sept4_il and HA-8xubiquitin, and treated with the proteasome inhibitor Lactacystin. Immunoprecipitation was done with either anti-ARTS or anti-Sept4_i1 antibody followed by *In vivo* ubiquitination assay. Western blot analysis was performed with anti-ubiquitin antibody. Parkin selectively ubiquitinates ARTS but not Sept4_i1, the non- apoptotic splice variant of *Sept4*.
(TIF)

Figure S2 **ARTS co-localizes with Parkin.** The distribution of ARTS and Parkin in HeLa cells was imaged by immunofluorescence before and at various times after treatment with Staurosporine (STS). **A.** In non-treated cells ARTS (green) is localized to mitochondria as shown by its typical punctated staining and co-localization with Mitotracker (red). After30 and 180 minutes of treatment with STS, ARTS increasingly exhibited diffused staining typical of cytosolic localization. **B.** In living cells, Parkin (green) staining shows a primarily cytosolic localization. Upon treatment with STS, Parkin immuno-reactivity can be seen at the mitochondria. **C.** In living cells, ARTS and Parkin are mainly showing mitochondrial and cytosolic pattern of staining, respectively. However, upon treatment with STS extensive co-localization of the two proteins was observed.
(TIF)

Table S1 **Induction of cell death in substantia nigra pars compacta (SNpc) with 6-OHDA results in increased levels of ARTS/caspase-3 relative to negative control.** Numbers of ARTS/cleaved caspase-3 double positive cells and ARTS/TUNEL double positive cells in SNpc area of 6-OHDA and saline treated rats are shown as percent of total number of cells (about 300) in microscope field. Counts were performed after one, three and seven days following injection. ***P<0.001, **P<0.01 for comparison with same day control by ANOVA followed by Bonferroni post hoc test.
(DOCX)
