# Supplementary figures and images for: Parkin Promotes Degradation of the Mitochondrial Pro-Apoptotic ARTS Protein
Source: PLoS One. 2012 Jul 9;7(7):e38837. doi: 10.1371/journal.pone.0038837 (PMC3392246; doi:10.1371/journal.pone.0038837)

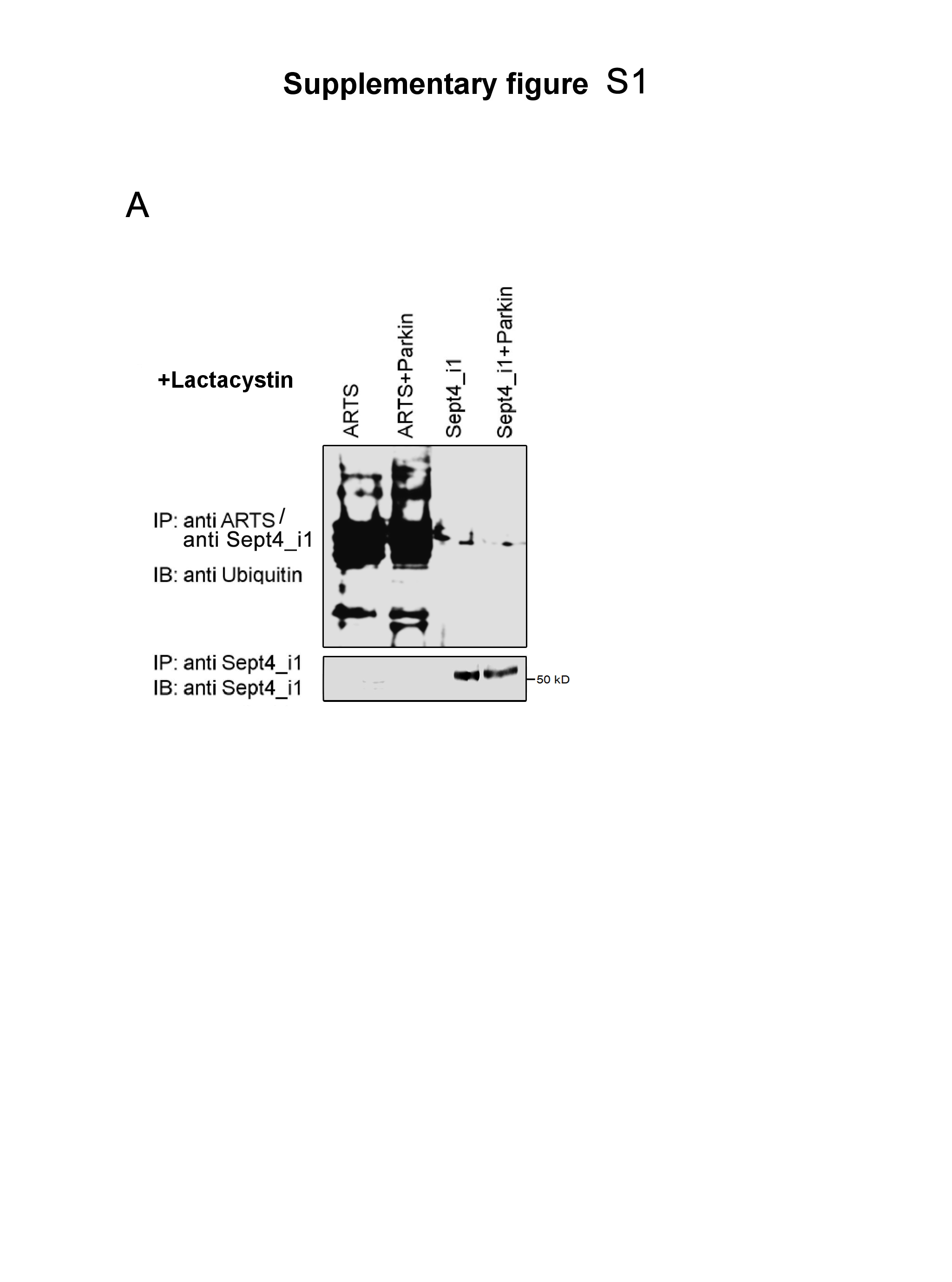

Supplement: Figure S1 — Parkin selectively ubiquitinates ARTS but not Sept4_i1. COS-7 cells were co-transfected with 1myc-Parkin together with 6myc-ARTS or Flag -Sept4_il and HA-8xubiquitin, and treated with the proteasome inhibitor Lactacystin. Immunoprecipitation was done with either anti-ARTS or anti-Sept4_i1 antibody followed by In vivo ubiquitination assay. Western blot analysis was performed with anti-ubiquitin antibody. Parkin selectively ubiquitinates ARTS but not Sept4_i1, the non- apoptotic splice variant of Sept4. (TIF) [file pone.0038837.s001.tif]

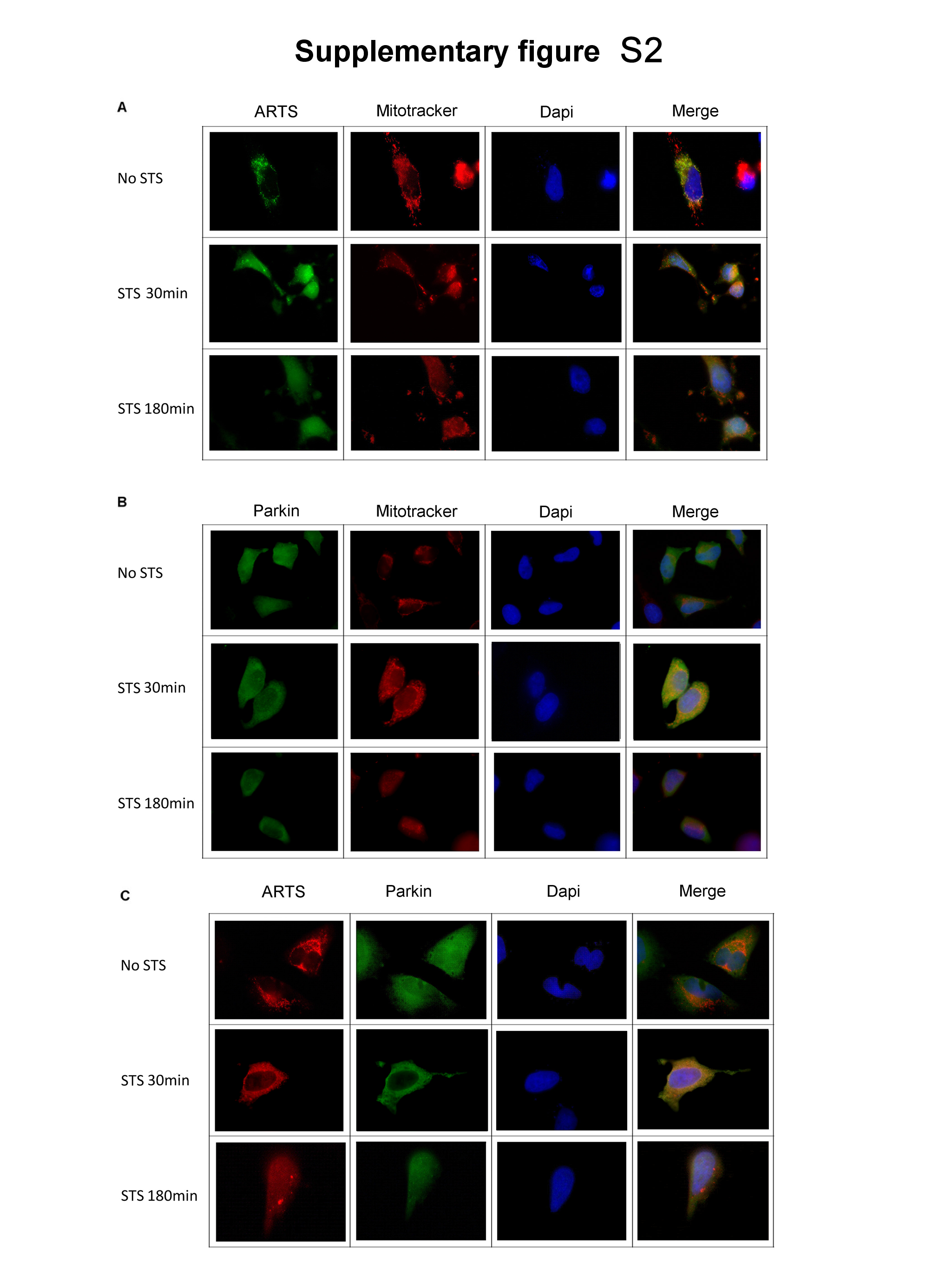

Supplement: Figure S2 — ARTS co-localizes with Parkin. The distribution of ARTS and Parkin in HeLa cells was imaged by immunofluorescence before and at various times after treatment with Staurosporine (STS). A. In non-treated cells ARTS (green) is localized to mitochondria as shown by its typical punctated staining and co-localization with Mitotracker (red). After30 and 180 minutes of treatment with STS, ARTS increasingly exhibited diffused staining typical of cytosolic localization. B. In living cells, Parkin (green) staining shows a primarily cytosolic localization. Upon treatment with STS, Parkin immuno-reactivity can be seen at the mitochondria. C. In living cells, ARTS and Parkin are mainly showing mitochondrial and cytosolic pattern of staining, respectively. However, upon treatment with STS extensive co-localization of the two proteins was observed. (TIF) [file pone.0038837.s002.tif]
