# Supplementary material for: Parkin Promotes Degradation of the Mitochondrial Pro-Apoptotic ARTS Protein
Source: PLoS One. 2012 Jul 9;7(7):e38837. doi: 10.1371/journal.pone.0038837 (PMC3392246; doi:10.1371/journal.pone.0038837)
Supplement: Table S1 — Induction of cell death in substantia nigra pars compacta (SNpc) with 6-OHDA results in increased levels of ARTS/caspase-3 relative to negative control. Numbers of ARTS/cleaved caspase-3 double positive cells and ARTS/TUNEL double positive cells in SNpc area of 6-OHDA and saline treated rats are shown as percent of total number of cells (about 300) in microscope field. Counts were performed after one, three and seven days following injection. ***P<0.001, **P<0.01 for comparison with same day control by ANOVA followed by Bonferroni post hoc test. (DOCX) [file pone.0038837.s003.docx]

**Supplementary table 1**

|  | **6-OHDA** | | | **Saline** | | |
| --- | --- | --- | --- | --- | --- | --- |
| **Days after treatment** | **1** | **3** | **7** | **1** | **3** | **7** |
| **ARTS/Caspase 3 cells (average)** | **7.71±1.79** | **1.97±1.27** | **0.57±0.12** | **1.47±0.88** | **1.3±0.71** | **0.88±0.27** |

|  | **6-OHDA** | | | **Saline** | | |
| --- | --- | --- | --- | --- | --- | --- |
| **Days after treatment** | **1** | **3** | **7** | **1** | **3** | **7** |
| **ARTS/TUNEL cells (average)** | **2.08±0.48** | **0.97±0.19** | **1.16±0.23** | **0.33±0.29** | **0.36±0.22** | **0.49±0.18** |

**Supplementary Table 1. Treatment of basal ganglia cells with 6OHDA results increased levels of ARTS/caspase-3 relative to negative control.** Numbers of ARTS/cleaved caspase-3 double positive cells and ARTS/TUNEL double positive cells in SNpc area of 6-OHDA and saline treated rats are shown as percent of total number of cells (about 300) in microscope field. Counts were performed after one, three and seven days following injection. *** P<0.001, ** P<0.01 for comparison with same day control by ANOVA followed by Bonferroni post hoc test.
